# Case Report of a Child with Colocolic Intussusception with a Primary Lead Point

**DOI:** 10.21980/J8564Q

**Published:** 2024-01-31

**Authors:** Ethan Lee, Jeremy Lins, Chelsea Cosand, Mary Jane Piroutek, Tommy Y Kim

**Affiliations:** * Loma Linda University School of Medicine, Loma Linda, CA; ^ HCA Healthcare, Riverside Community Hospital, Department of Emergency Medicine, Riverside, CA; † Children’s Hospital of Orange County, Department of Pediatric Emergency Medicine, Orange, CA

## Abstract

Intussusception is the telescoping of bowel into an adjacent segment of bowel and has an associated risk for bowel ischemia and perforation. The classic triad of abdominal pain, blood in stool, and an abdominal mass is present in less than 40% of pediatric cases and is less common in older children.^1^ Ultrasound has a high sensitivity and specificity for the diagnosis of intussusception, and once diagnosed, treatment modalities include reduction by either ultrasound or fluoroscopic guided air or hydrostatic enema. The risk of recurrence after successful reduction occurs in up to 12% of pediatric patients and occurs more frequently in older children and children with a pathologic lead point.^2^ We present a case of a 6-year-old child with colocolic intussusception that was successfully reduced and recurred within five days due to a large colonic polyp.

**Topics:**

Intussusception, lead point, pediatrics.

[Fig f1-jetem-9-1-v15] [Fig f2-jetem-9-1-v15] [Fig f3-jetem-9-1-v15]

**Figure f1-jetem-9-1-v15:**
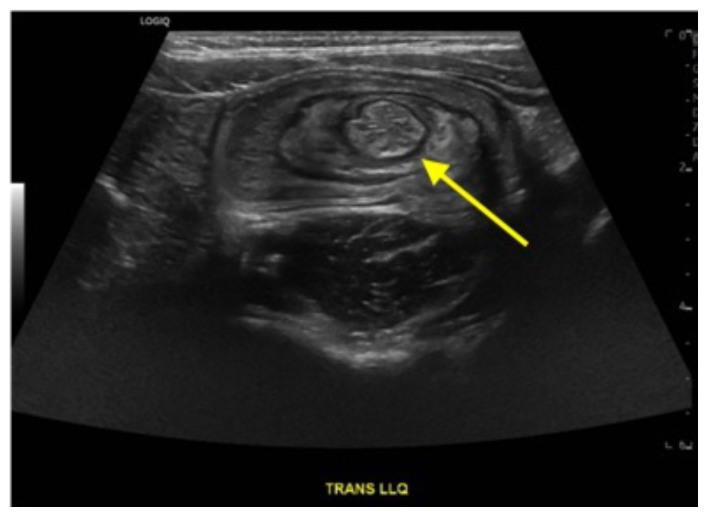


**Figure f2-jetem-9-1-v15:**
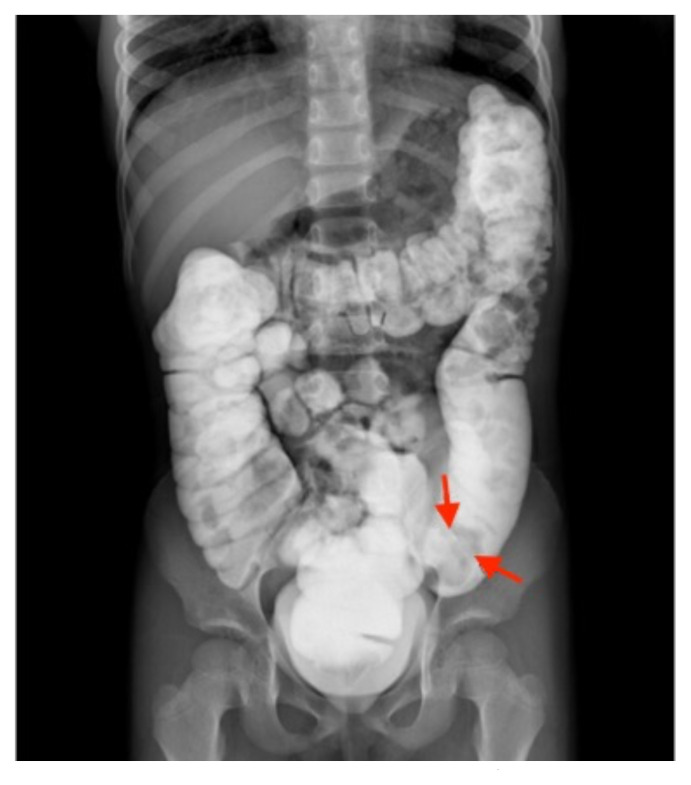


**Figure f3-jetem-9-1-v15:**
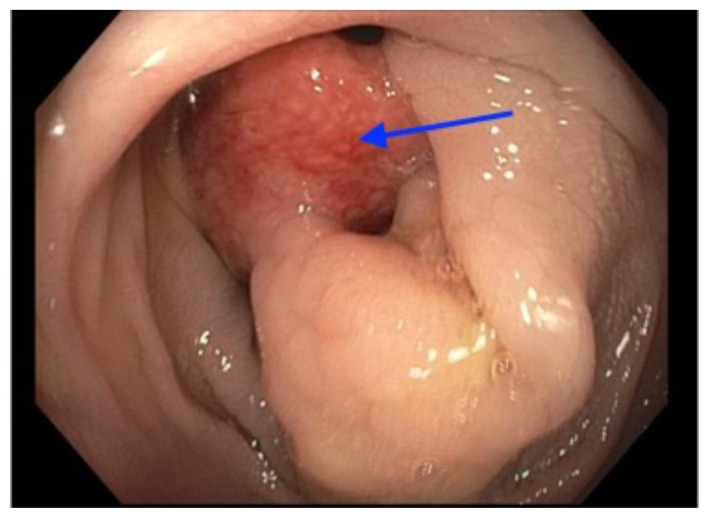


## Brief introduction

Intussusception is the telescoping of one segment of bowel into an adjacent segment of bowel and is the second leading cause of bowel obstruction in the children less than two years of age.^3^ Ileocolic intussusception is the telescoping of the ileum into the colon and is the most common type of intussusception seen in pediatric patients, accounting for 90% of cases.^2^ Cases involving the telescoping of colon into colon, colocolic intussusception, are rare and contribute up to 3% of all cases of pediatric intussusception.^4^

The mean age for intussusception is 6–18 months with only 30% of cases occurring over the age of 2 years.^3^ Although most cases of intussusception in children are benign, it can lead to intestinal ischemia and/or perforation if not treated immediately.^5^–^6^ The majority of ileocolic intussusception cases are idiopathic. Pathologic lead points are more likely to be found in older pediatric patients and in patients with colocolic intussusception. The most common pathologic lead point is a Meckel’s diverticulum. Other lead points include intestinal polyps, intestinal duplication, allergic purpura, and tumors.^7^ Risk factors associated with a pathologic lead point include older age, position, diameter and length of the intussusception, and the presence of free intra-abdominal fluid.^7^

## Presenting concerns and clinical findings

A 6-year-old healthy female who recently emigrated from Southeast Asia presented to a community emergency department (ED) with abdominal pain and blood-streaked loose stools. The abdominal pain had been present for 1 week and was described as cramping and colicky occurring multiple times a day. When severe, the patient would curl into a fetal position for comfort, but between episodes, the patient was completely pain free. Blood-streaked loose stools began on the day prior to the ED visit. The patient had no past medical or surgical history. A focused review of systems revealed anorexia with 6 episodes of diarrhea in the past 24 hours with no fever or vomiting. The patient’s presenting vital signs were within normal limits for age. Her initial physical examination was benign with a soft, non-tender, non-distended abdomen with no palpable masses and normal bowel sounds. Her external anal examination demonstrated no fissure or hemorrhoids. While in the ED, the patient had an episode of abdominal pain with generalized tenderness and no palpable masses. Her pain resolved spontaneously within 15 minutes, and her repeat abdominal examination after the episode was again benign.

## Significant findings

On the initial ED visit, an abdominal ultrasound (US) was ordered which showed the classic intussusception finding of a target sign (yellow arrow), or concentric rings of telescoped bowel, on the transverse view of the left lower quadrant (LLQ). The patient was transferred to a local tertiary pediatric hospital where a fluoroscopic enema demonstrated a reduced intussusception, and she was discharged home. Within a week, the patient presented to the community ED with recurrent symptoms and an US revealed a recurrent intussusception. The patient was transferred again to the tertiary pediatric hospital, and a repeat fluoroscopic enema demonstrated reduction of the intussusception. However, a persistent filling defect, shown by disruption of the normal filling of the colon cavity, was found in the LLQ (red arrows).

## Patient course

The patient was admitted, and a colonoscopy was recommended by the pediatric gastroenterologist. The colonoscopy revealed a large descending colonic polyp (blue arrow) as a pathologic lead point for the intussusception. The polyp was removed, and pathology revealed normal glandular architecture with small lymphoid aggregate in the lamina propria without dysplasia or malignancy. The patient was discharged home after several days with no complications.

## Discussion

The classic clinical triad of intermittent abdominal pain, currant jelly stool, and a sausage shaped abdominal mass are present in less than 40% of intussusception cases.^1^ In younger children less than 24 months of age, findings of abdominal pain, vomiting, lethargy, rectal bleeding, and irritability were found to be predictors for intussusception when compared to children greater than 24 months of age.^4^.

Imaging plays a major role in the diagnosis of intussusception. Typical imaging strategies include radiography and ultrasound. Radiographs have a diagnostic accuracy for ileocecal intussusception of approximately 25% and carry a high false negative rate.^8^ Findings can include signs of obstruction with air fluid levels and/or dilated loops of bowel, an absent liver margin, or lack of air in the cecum.^3^,^9^ Intussusception on radiographs can also reveal a crescent sign, which is a semicircular lucency created by gas trapped between two intussuscepted bowel walls.^8^

Ultrasound has a high sensitivity and specificity of 92–100% and has become the imaging modality of choice for the diagnosis of intussusception because of its high specificity and sensitivity as well as the speed and ease of use.^8^–^10^ Ultrasound findings of intussusception include the classic finding on transverse view of a target or donut sign which are made of concentric rings. On the longitudinal view, parallel stripes with different echogenicity can show a pseudokidney or sandwich sign.^8^,^9^ In a meta-analysis, the emergency physician’s ability in diagnosing ileocecal intussusception using point of care ultrasound was found to have a 94.9% (95% CI, 89.9% to 97.5%) sensitivity and a 99.1% (95% CI, 94.7% to 99.8%) specificity.^10^

The treatment of intussusception is reduction by either ultrasound or fluoroscopic guided air or hydrostatic enema.^3^ Surgical intervention is required with failed attempts at enema reduction. In a meta-analysis comparing different reduction techniques, Plut, et al, found fluoroscopic-hydrostatic enema reduction had a 67% success rate with a perforation rate of 2%, fluoroscopic-pneumatic enema had an 81% reduction rate and 1% perforation rate, ultrasound-guided hydrostatic enema had 82% success rate and 1% perforation rate, and ultrasound-guided pneumatic enema had a 93% reduction rate and 1% perforation rate. Although ultrasound-guided pneumatic enema was found to have the best reduction rate, the disadvantage of difficult visualization of intussusception during the procedure as well as visualization of the reduction limit its widespread use.^9^

After successful reduction, recurrence occurs at a rate of 8% to 12%.^2^ In a national database review, Ferrantella found a 30-day recurrence rate of 3.7% for non-operative reduction, 2.3% for surgical reduction, and 0% for cases with bowel resection. Recurrence was more frequent in the non-operative group with a median time for readmission of four days, and only 1.5% recurred within 48 hours of discharge.^2^ Guo, et al, found risk factors associated with recurrence was age greater than one year, duration of symptoms less than 12 hours, absence of vomiting, mass location, and a pathologic lead point. In the presence of a lead point, vomiting and mass location (left abdomen) were predictive of recurrence.^11^ In a 2021 systematic review, twelve studies compared complication rates of patients admitted for observation versus discharge home from the ED after successful reduction. There was no difference in the overall recurrence rates of 8.8% for ED discharge versus 8.5% for inpatient observation. Children greater than two years of age were found to be the only predictor for recurrence after discharge.^12^

Colocolic intussusception is far less common than ileocolic intussusception in pediatric patients.^4^ The majority of colocolic intussusception is the result of a pathologic lead point, of which polyps are most common.^13^ In a case series of colocolic intussusception, Richer compared colocolic with ileocolic intussusception and found enema reduction to be successful in 33% of colocolic cases compared to 67% for ileocolic cases. They also found that 25% of colocolic intussusception required colonic resection due to ischemia in comparison to 9% for ileocolic intussusception.^4^

Although the patient presented with abdominal pain and blood in her stool, her initial examination was benign with no palpable masses and no finding of a surgical abdomen. At the time of diagnosis, the ultrasound found an intussusception in the LLQ of the abdomen, suggesting a diagnosis of a colocolic intussusception which increased the risk for recurrence as well as a pathologic lead point After the patient returned with recurrent intussusception, colonoscopy did reveal a large polyp which was removed. Emergency providers should have a lower threshold for admission of children with colocolic intussusception, given the higher risk of recurrence and the risk of a pathologic lead point.

## Disclaimer Statement

This research was supported (in whole or in part) by HCA Healthcare and/or an HCA Healthcare affiliated entity. The views expressed in this publication represent those of the author(s) and do not necessarily represent the official views of HCA Healthcare or any of its affiliated entities.

## Supplementary Information












