# Supplementary figures and images for: Case Report of a Child with Colocolic Intussusception with a Primary Lead Point
Source: J Educ Teach Emerg Med. 2024 Jan 31;9(1):V15–8. doi: 10.21980/J8564Q (PMC10854881; doi:10.21980/J8564Q)

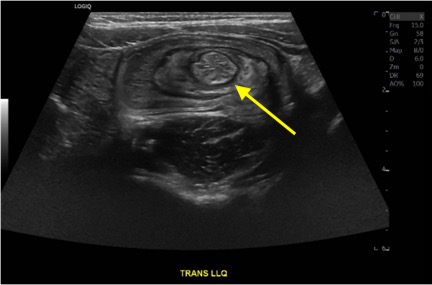

Supplement: Supplementary file 1 [file jetem-9-1-V15-supp1.jpg]

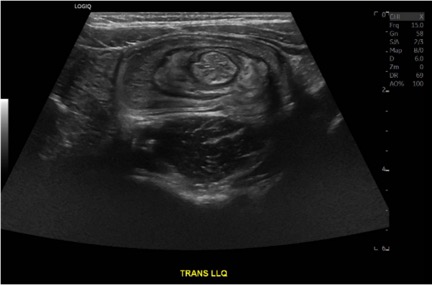

Supplement: Supplementary file 2 [file jetem-9-1-V15-supp2.jpg]

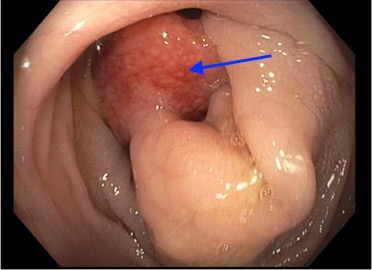

Supplement: Supplementary file 3 [file jetem-9-1-V15-supp3.jpg]

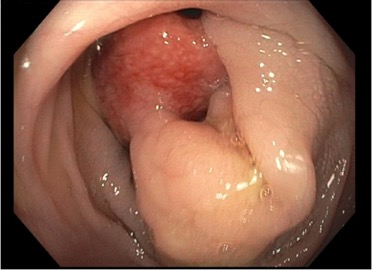

Supplement: Supplementary file 4 [file jetem-9-1-V15-supp4.jpg]

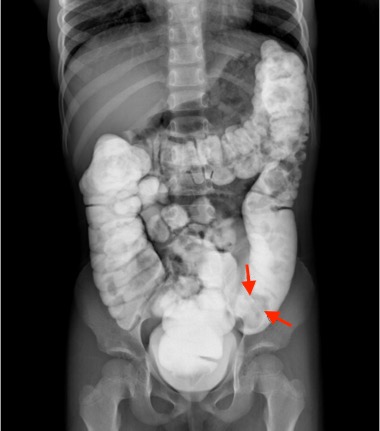

Supplement: Supplementary file 5 [file jetem-9-1-V15-supp5.jpg]

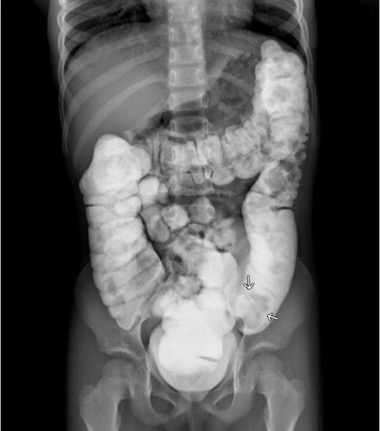

Supplement: Supplementary file 6 [file jetem-9-1-V15-supp6.jpg]
